# Shear wave ultrasound elastography of the biceps brachii can be used as a precise proxy for passive elbow torque in individuals with hemiparetic stroke

**DOI:** 10.14814/phy2.15691

**Published:** 2023-05-19

**Authors:** Michael D. Ellis, Netta Gurari, Ninette T. A. Gerritsen, Sabrina M. Lee, Amy Wang, Julius P. A. Dewald

**Affiliations:** ^1^ Department of Physical Therapy and Human Movement Sciences Northwestern University Chicago Illinois USA; ^2^ Department of Mechanical Engineering Northwestern University Evanston Illinois USA; ^3^ Mechanical, Maritime and Materials Engineering (3mE) Delft University of Technology Delft Netherlands; ^4^ Department of Physical Medicine and Rehabilitation Northwestern University Chicago Illinois USA; ^5^ Department of Biomedical Engineering Northwestern University Chicago Illinois USA

**Keywords:** criterion validity, joint torque, mechatronics, stroke, torque, ultrasound elastography

## Abstract

Muscle tissue is prone to changes in composition and architecture following stroke. Changes in muscle tissue of the extremities are thought to increase resistance to muscle elongation or joint torque under passive conditions. These effects likely compound neuromuscular impairments, exacerbating movement function. Unfortunately, conventional rehabilitation is devoid of precise measures and relies on subjective assessments of passive joint torques. Shear wave ultrasound elastography, a tool to measure muscle mechanical properties, may be readily available for use in the rehabilitation setting as a precise measure, albeit at the muscle‐tissue level. To support this postulation, we evaluated the *criterion* validity of shear wave ultrasound elastography of the biceps brachii; we investigated its relationship with a laboratory‐based criterion measure for quantifying elbow joint torque in individuals with moderate to severe chronic stroke. Additionally, we evaluated *construct validity*, with the specific sub‐type of *hypothesis testing of known groups,* by testing the difference between arms. Measurements were performed under passive conditions at seven positions spanning the arc of elbow joint flexion‐extension in both arms of nine individuals with hemiparetic stroke. Surface electromyography was utilized for threshold‐based confirmation of muscle quiescence. A moderate relationship between the shear wave velocity and elbow joint torque was identified, and both metrics were greater in the paretic arm. Data supports the progression toward a clinical application of shear wave ultrasound elastography in evaluating altered muscle mechanical properties in stroke, while acknowledging that undetectable muscle activation or hypertonicity may contribute to the measurement. Shear wave ultrasound elastography may augment the conventional method of manually testing joint mobility by providing a high‐resolution precise value. Tissue‐level measurement may also assist in identifying new therapeutic targets for patient‐specific impairment‐based interventions.

## Graphical abstract

Precise and objective methods for quantifying passive joint torques are absent in stroke rehabilitation, leaving rehabilitation specialists to subjectively assess its impact on function. Here, we compare shear wave ultrasound elastography of the biceps brachii muscle with a mechatronic method (criterion measure) of measuring elbow flexion torque over a range of elbow angles. Differences are evaluated between arms. Data support the clinical application of shear wave ultrasound elastography, especially in the absence of bedside mechatronics.

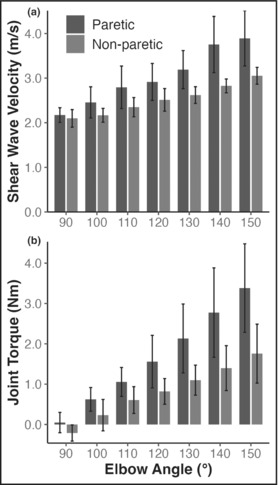

## 1 INTRODUCTION

Methods for precise and objective measurement of resistance to passive range of motion are absent in the stroke rehabilitation setting leaving clinicians to low resolution, subjective ordinal clinical scales. This is problematic for clinical decision‐making that attempts to prioritize the underlying contributing factors to movement problems that should be addressed with intervention. It is even more important in the later stage of stroke recovery that is notable for a loss of independent joint control (Dewald et al., 2001), hypertonia (Burke et al., 2013), and muscle shortening (Nelson et al., 2018) which affects reaching range of motion (Sukal et al., 2007). The diminished activity and use of the arm (Ellis et al., 2008) that accompanies the loss of independent joint control places patients at risk for secondary musculoskeletal changes such as muscle atrophy (English et al., 2010), reduced fascicle length (Li et al., 2007), loss of sarcomeres in series (Adkins et al., 2021) and accumulation of connective tissue or muscle fibrosis, increasing passive joint torques (Lieber & Ward, 2013). These muscle tissue changes threaten to compound neuromuscular impairments of reaching range of motion, like loss of independent joint control and hypertonia, that results in decreased elbow extension excursion and movement velocity due to the resistance imparted by abnormal flexor activation (Ellis et al., 2017). In fact, the passive mechanical contributions of resistance to joint rotation, or passive joint torques, are increased at the ankle (Chung et al., 2004; Gao et al., 2009; Given et al., 1995; Lorentzen et al., 2010; Magnusson, 1998; Mirbagheri et al., 2004, 2007) and elbow (Alibiglou et al., 2008; Given et al., 1995; McCrea et al., 2003; Mirbagheri et al., 2004, 2007) in individuals with stroke. More specifically and relevant to reaching function, resistance to passive elbow extension increases toward the end of the range of motion (Alibiglou et al., 2008; Chino & Takahashi, 2015; Magnusson, 1998; Mirbagheri et al., 2007, 2009). Importantly, the joint‐level impairment reflects the tissue‐level exponential passive stress‐strain relationship that is reported for muscle fascicles (Abbott & Aubert, 1951; Rassier et al., 2005; Rehorn et al., 2014) and whole muscle (Persad et al., 2021), highlighting the role of muscle tissue stiffness in passive joint torque. Unfortunately, methods for the measurement of passive joint torque or resistance to movement that would benefit clinical decision‐making, have been restricted to laboratory‐based investigations, limiting clinicians to a subjective manual evaluation of joint mobility and end‐feel (Page et al., 2010). Objective tools for clinical evaluation are needed to improve rehabilitation diagnoses of physiological factors contributing to reaching dysfunction following stroke.

Clinical evaluation at the musculoskeletal level is an alternative approach and may provide more precise mechanistic information; therefore, can identify potential therapeutic targets for the development of impairment‐based rehabilitation interventions. The measurement of passive joint torque represents the net effect of all tissues surrounding the joint including, but not limited to muscles, ligaments, and joint capsules (Riemann et al., 2001); however, muscle tissue is thought to be the major contributor (Lieber et al., 2017). Integrating interventions targeting stroke‐related muscle tissue changes may augment current impairment‐based interventions that are aligned with restoring premorbid neural control of movement. For example, the prominent expression of a flexion synergy and impaired reaching range of motion in severe stroke (Ellis et al., 2008) suggests that elbow flexors may be the primary contributor to joint‐level changes in passive torque. Therefore, targeting stroke‐related changes in biceps brachii may augment interventions for the flexion synergy, such as progressive abduction loading therapy (Ellis et al., 2018). Successful administration of this approach would require evaluation at the tissue level. However, until recently, the only technique available to quantify the mechanical properties of individual muscles is magnetic resonance elastography (Litwiller et al., 2012), which is too costly to justify in stroke rehabilitation.

A prime candidate for the measurement of mechanical properties at the muscle tissue level in the clinical setting is shear wave ultrasound elastography. Shear wave ultrasound elastography is a promising tool to enhance the diagnostic capabilities of rehabilitation specialists as it is routinely utilized in conventional practice for imaging other tissues such as breast (Barr et al., 2015) and liver (Ferraioli et al., 2015). Shear wave ultrasound elastography can be employed using a conventional diagnostic ultrasound device equipped with shear wave capabilities to measure shear wave velocity in muscle (Lacourpaille et al., 2012). Shear wave velocity is proportional to the square root of the shear modulus and, therefore, is often reported as a surrogate measure, or proxy, for the elastic properties of biological tissue (Bercoff et al., 2004; Chino et al., 2012; Eby et al., 2013). Evidence for validity of shear wave velocity as a surrogate measure of muscle properties in passive muscle has been demonstrated in cadaveric animal preparations, demonstrating a relationship of shear elastic modulus with muscle force (Koo et al., 2013) and muscle stiffness (Young's modulus) (Eby et al., 2013). Recent in situ feline studies have further differentiated that shear wave velocity is sensitive to both muscle force and force‐dependent changes in muscle stiffness (Bernabei et al., 1985, 2021).

Shear wave ultrasound elastography has been routinely employed to evaluate the effects of hemiparesis, in both cerebral palsy (Kwon et al., 2012) and stroke (Lee et al., 2015, 2016), with systematic reviews supporting its reliability and validity (Lehoux et al., 2020; Miller et al., 2021; Zuniga et al., 2021). Evidence for the validity of ultrasound elastography primarily involves comparisons with low‐resolution, ordinal clinical impairment scales (Miller et al., 2022). Specifically, for the evaluation of *criterion* validity (following COSMIN terminology) (Mokkink et al., 2010), the use of a precise mechatronic criterion measure (joint torque) exists for neurologically intact humans (Chino & Takahashi, 2015) and individuals with stroke for both the ankle joint under passive conditions (Jakubowski et al., 2017) and the elbow joint under active conditions (Lee et al., 2019). Additional evidence supporting the *criterion* validity of ultrasound elastography in hemiparetic biceps was conducted by Eby et al. (2016); however, the results were limited in that half of the sample (*n* = 4 of 9) were mildly impaired such that they presented similarly to controls. Taken together, these studies warrant further investigation of the criterion validity by implementing a more precise criterion, for example, joint torque, in the elbow joint under passive conditions. This is especially necessary for individuals with homogeneous moderate/severe impairment.

Therefore, the primary aim of this study was to evaluate the *criterion* validity of ultrasound elastography for assessing the physiological factors underlying resistance to passive range of motion following stroke. The relationship between shear wave velocity of biceps brachii, as measured using ultrasound elastography and elbow extension joint torque was investigated using a custom one‐degree‐of‐freedom device (Euving et al., 2016) at seven angular positions. The focus on a single muscle for ultrasonography may present a limitation in that between‐muscle differences in shear modulus have been reported in the ankle joint of individuals with stroke (Le Sant et al., 1985). However, there was no difference between the paretic brachioradialis and biceps brachii in another study (Galvao et al., 2022). Biceps brachii is also known to contribute only partially to the net joint torque exerted about the elbow joint (36% during isometrics and 48% during voluntary movements) (Tax et al., 1990), in comparison to brachialis (57% and 45%, respectively). In the present study, the rationale for a single muscle for ultrasonography is sensible in that a single measure is most likely to be efficient enough to translate to clinical practice. Joint torque was chosen as the criterion measure, instead of alternative criterion measures, such as magnetic resonance elastography as it represents a precise analogue to what is manually performed by the clinician when evaluating passive joint mobility. We hypothesized that there would be a positive/direct relationship between the two measurement techniques supporting its *criterion* validity. We also investigated the difference between paretic and non‐paretic arms in an important effort to replicate prior findings discussed above and further support the *construct* validity of ultrasound elastography in evaluation of individuals with stroke. The results of this investigation provide evidence for both the *criterion* and *construct* validity (*hypothesis testing of known groups*) of ultrasound elastography in hemiparetic stroke muscle and support its application in clinical practice.

## 2 MATERIALS AND METHODS

### 2.1 Participants

A total of 12 individuals (six men, six women) age 57 ± 10 years with chronic stroke (13 ± 9 years post) and upper extremity Fügl‐Meyer Motor Assessment scores of 27 ± 3 out of 66, participated in the study. All participants were recruited from the Clinical Neuroscience Research Registry of Shirley Ryan AbilityLab and the Department of Physical Therapy and Human Movement Sciences of Northwestern University. The inclusion criteria for individuals were: (1) hemiparetic stroke with a moderate to severe impairment of the upper limb as determined by clinical evaluation using the Arm Motor Fügl‐Meyer Assessment, (2) adequate passive range of motion at the shoulder and elbow joints without pain at end range, (3) ability to provide informed consent, and (4) greater than 6 months since their stroke.

Prior to the experiment, all participants gave informed consent to participate in the study. The study was approved by the Institutional Review Board of Northwestern University in accordance with the ethical standards laid down in the 1964 Declaration of Helsinki for research involving human subjects.

Participants were first screened by a licensed physical therapist (Ellis) to confirm pain‐free passive range of motion at the shoulder and elbow. The shoulder was required to have passive abduction (frontal plane elevation) to at least 100°, and the elbow was required to have a passive extension to at least 30° less than full normal range without pain at end range with over‐pressure. This was not an issue as all participants had full passive elbow extension. No participants were excluded for conditions such as: (1) pain at end range with over‐pressure indicating inflammation or (2) current pain in the extremities or spine. However, two participants were ultimately excluded due to the inability to achieve quiescent muscle activity (see below in “protocol”), and one was excluded due to corrupted data. This reduced the study sample to *n* = 9.

### 2.2 Experimental setup

**FIGURE 1:**
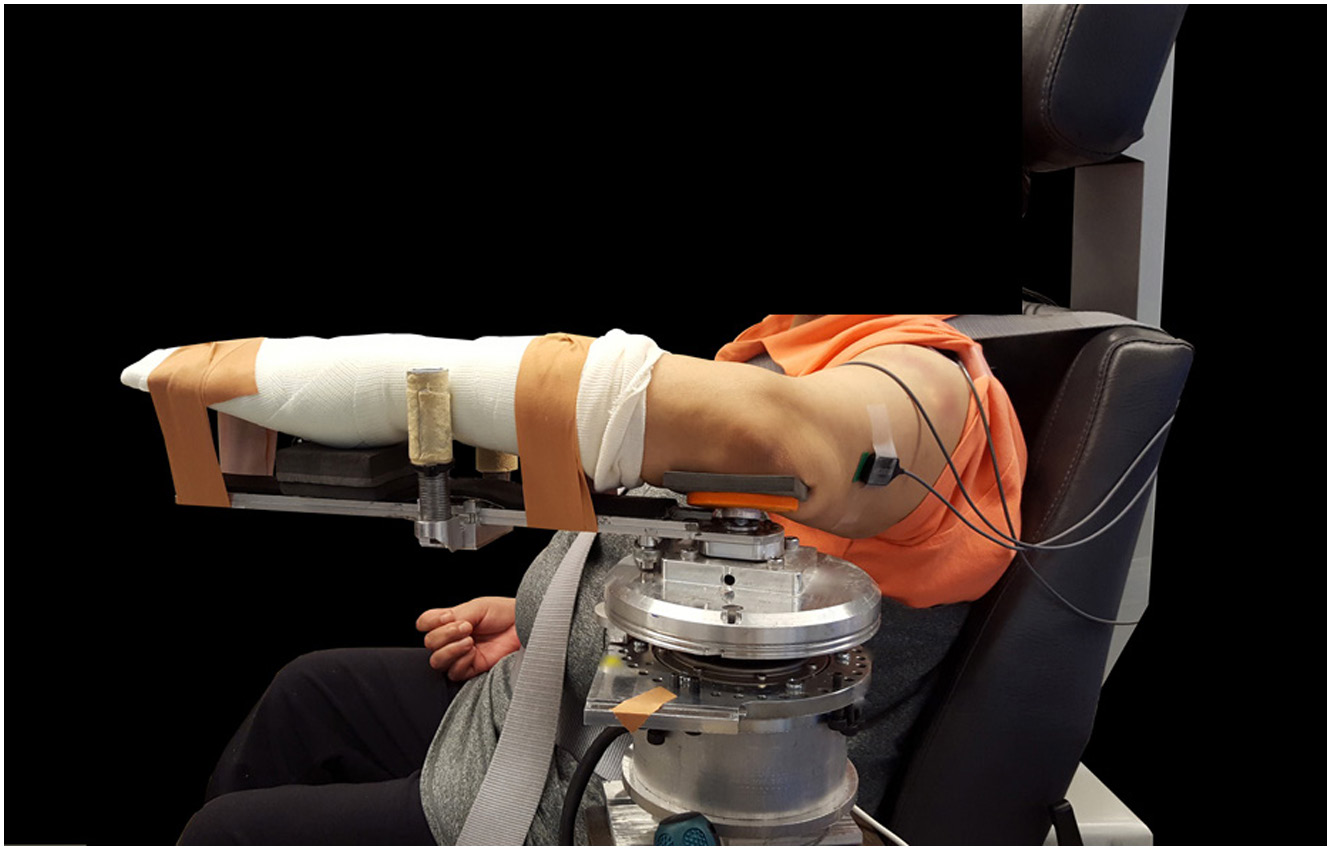
Experimental setup. The lower arm is casted and mounted comfortably to the swing arm of the robotic device in the standard arm configuration. The torso is secured to the seat with nylon strapping. EMG electrodes are in place on the biceps brachii and the long head of the triceps brachii.

The participant was positioned in the Biodex chair (Biodex Medical Systems, Inc.), with the arm/shoulder abducted 90°, arm/shoulder rotated 45° from the coronal plane, and elbow at 90° (Figure 1). The participant was strapped into the Biodex chair with nylon belts to constrain movement of the upper body and shoulder girdle. The forearm, including wrist and fingers, were casted with fiberglass and indirectly coupled to a one‐degree‐of‐freedom force sensor with a resolution of <0.005 N and force range of 0‐300 N (Stamford, Omega Engineering Inc., LCM201‐300) via a custom single‐degree‐of‐freedom robotic device (Euving et al., 2016). Data signals from the force sensor were read into the computer using a 16‐bit data acquisition board (Markham, ON, Canada, Quanser, Q8). The fiberglass casting allowed for a non‐invasive rigid coupling of the participant's forearm to the device, encouraging direct force transmission from the participant's forearm to the load cell. Casting also constrained the pronation/supination degree‐of‐freedom. The medial epicondyle was physically palpated and placed in the middle of a padded cup, such that the axis‐of‐rotation of the elbow was aligned with the axis‐of‐rotation of the robotic device. The robotic device controlled the elbow joint angular velocity and concurrently measured the torque about the elbow joint. An encoder (Peabody, US250), that was attached to the device's motor, measured the angular position of the elbow joint. Surface EMG (Delsys, Bagnoli EMG System) was recorded throughout the session for the biceps brachii and the long head of the triceps brachii. Active differential electrodes with a 1 cm inter‐electrode distance were used. EMG signals were pre‐filtered with a low‐pass cutoff frequency of 500 Hz (8‐pole Butterworth filter) and amplified by 1000. Torque/angle and EMG data were sampled at 1 kHz and stored offline for post hoc analysis. Shear wave velocity images were captured using an ultrasonography system (Aixplorer SuperSonic Imagine) with a linear transducer array (4–15 MHz, SuperLinear 15–4, Vermon). The parameters of the system were set to (i) mode: MSK—foot and ankle; (ii) opt: std; and (iii) persist: no, to maximize the sampling rate and avoid bias due to locked proprietary data post‐processing.

### 2.3 Protocol

The participant was first positioned in the standardized configuration as described in the experimental setup section. Once positioned, the elbow joint was manually flexed and extended by the experimenter over the entire range of motion to confirm proper alignment and a comfortable full range of motion at the elbow joint upon a stationary humerus. EMG sensors were placed on prepped skin over the medial muscle belly of the biceps brachii and the long head of the triceps brachii. The ground sensor was placed proximally over the acromioclavicular joint. The participant's elbow joint was then placed in a standardized angular position of 90°, and the software recorded this angular position as the 90° home position.

A standardized stretch reflex habituation procedure was then administered to minimize stretch reflex‐related muscle activity (Schmit et al., 2000). In this procedure, the elbow joint was passively rotated between 75° and 150° (elbow angle) at 120°/s for 20 repetitions. The angular velocity was sufficient to elicit repetitive stretch reflex activity, resulting in a reduction of abnormal tonic muscle activation (Patterson et al., 2022; Schmit et al., 2000). If muscle activation was present after the stretches, visualized as spikes in the steady‐state EMG signal while at rest, stretches were repeated. If quiescence was confirmed, a subsequent 5‐s recording was made and stored offline for future analysis to define the “baseline” quiescent EMG signal. As mentioned above, two participants were unable to achieve quiescence in their muscle activity leading to the termination of the experiment. After the habituation procedure, the torque signal was zeroed with the participant at rest in the standardized 90° elbow joint angular position.

**FIGURE 2:**
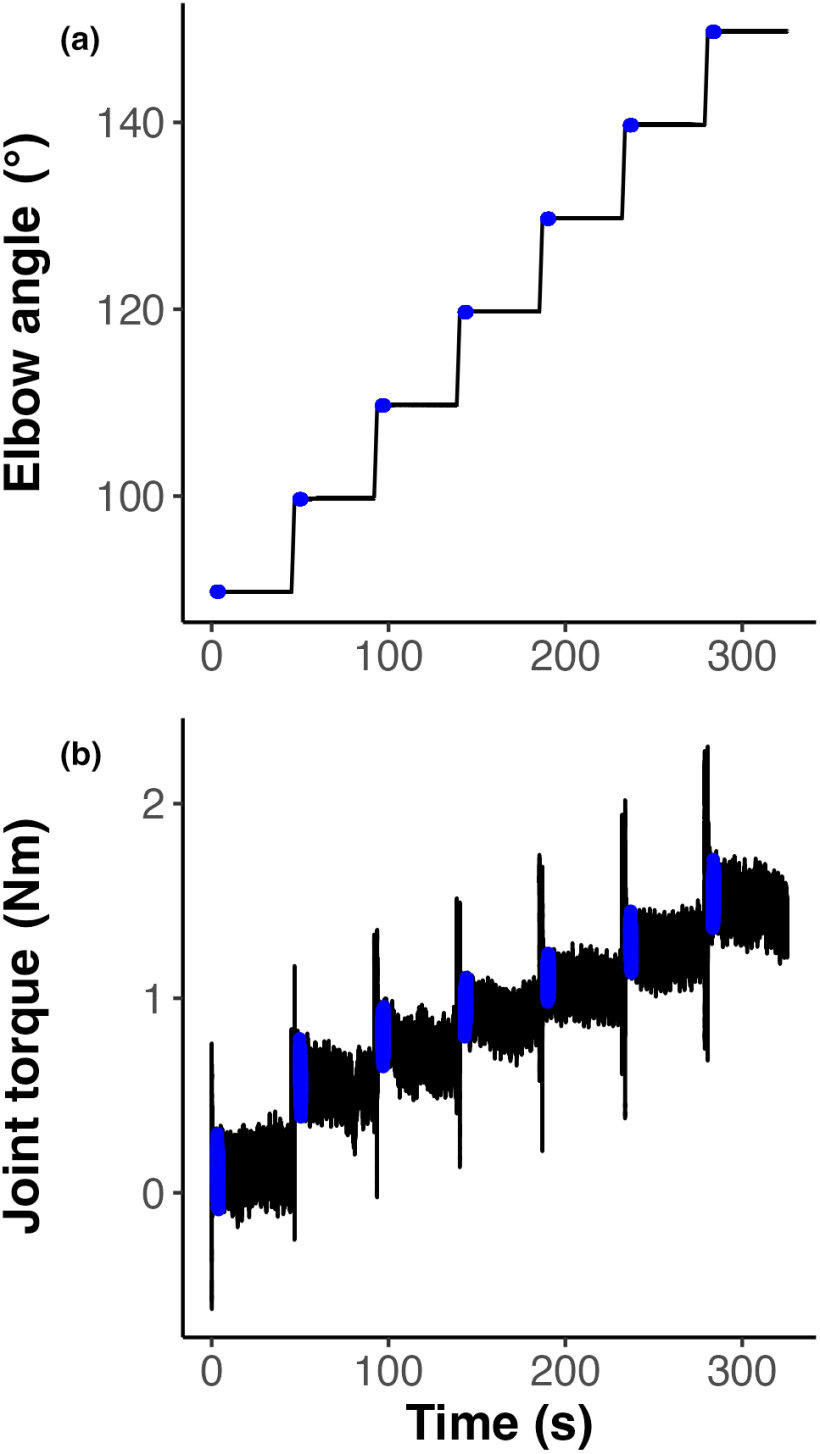
An example of continuous elbow angle (a) and joint torque (b) data over seven elbow joint positions (90°–150°), with the color blue indicating the 3.0–4.0 s time interval at the beginning of each joint position where joint torque was extracted occurring before when the ultrasound images were acquired.

**FIGURE 3:**
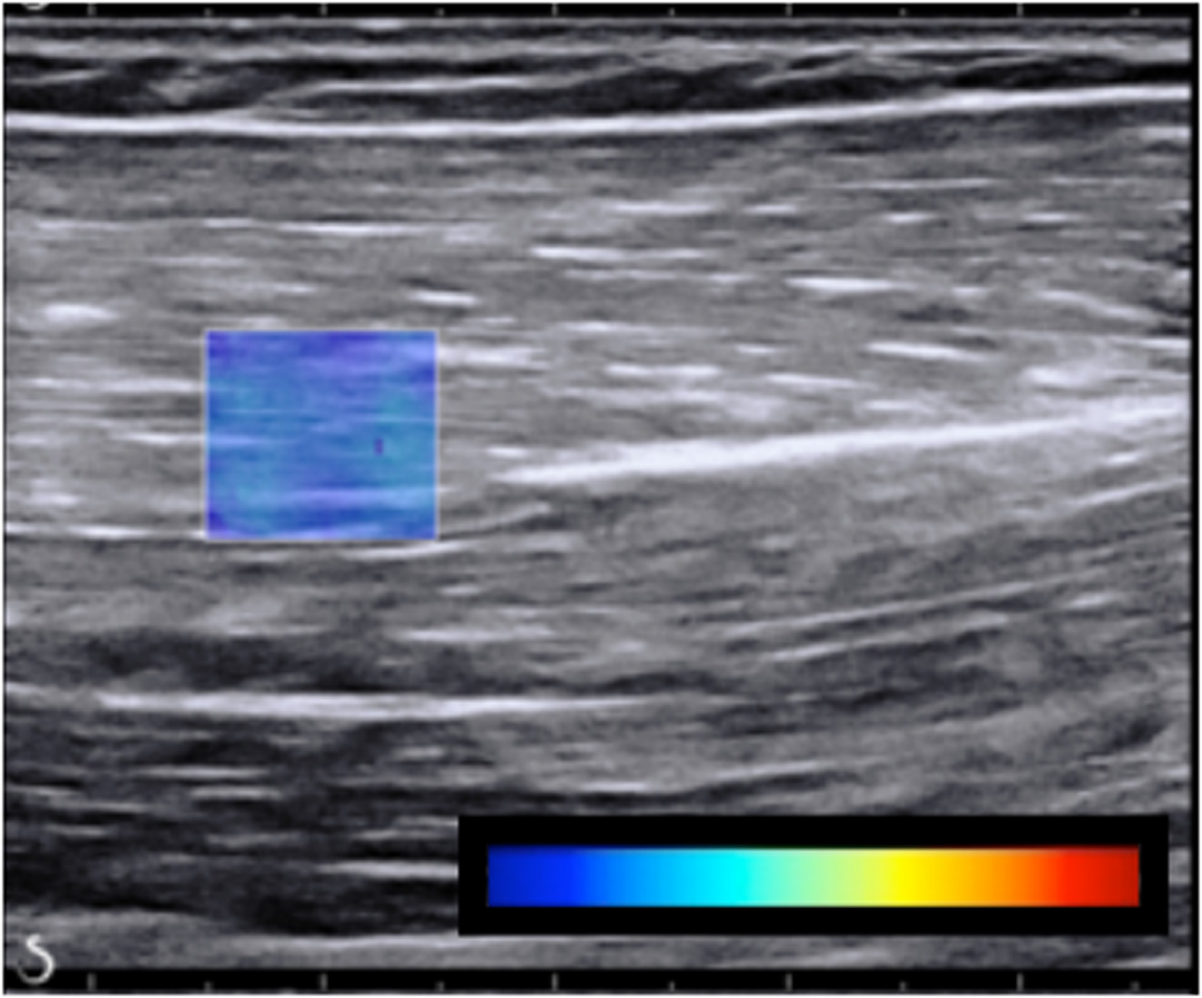
Shear wave ultrasound elastography image for the paretic biceps brachii at the 110° elbow angle position. The color‐coded box represents the region of interest, and the color bar illustrates velocity from 0 m/s (dark blue) to 8 m/s (red).

A standardized procedure was then administered for the acquisition of passive elbow joint torque and ultrasound elastography images of the biceps brachii at seven different elbow joint angle positions (Figure 2). In this procedure, the device rotated the elbow at a slow constant angular velocity of 6°/s to each of the seven positions in order to avoid stretch reflex‐related muscle activation (Schmit et al., 2000). The most acute elbow angle measured was 90° and proceeded with 100°, 110°, 120°, 130°, 140°, and 150°, representing measurements in the “extension” direction. Next, the direction was reversed, collecting the same data in the same angular positions but proceeding in the “flexion” direction. The 90° position was the most acute angle that could be measured due to the size of the ultrasound transducer and its proximity to adjacent anatomy. At each angular position, five images were collected that passed the following criteria: (1) muscle fascicles were aligned in the plane of the transducer, (2) the shear wave velocity map region of interest (box in Figure 3) was placed in the mid‐belly region distal and superficial to the central aponeurosis of the biceps, (3) the shear wave velocity map region of interest illustrated acquisition of velocity data throughout the entire area, (4) no muscle EMG activity was visually observed from either of the muscles, (5) minimal transducer pressure was applied to the muscle, and (6) images were captured 45 s after placing the joint in the subsequent angular position (Kot et al., 2012). Visual inspection of the electromyography signal for muscle activation and participant posture was performed throughout the experiment. An output signal from the ultrasound system indicating image capture was recorded with the torque, angular position, and EMG data; the EMG signals were analyzed offline to confirm muscle quiescence when the ultrasound data were captured.

The final step in the protocol was to determine maximum voluntary muscle activation. This served the sole purpose of providing a frame of reference for the determination of quiescence discussed below in the data analysis section. In this procedure, the participant's forearm was taken out of the robotic device. The participant remained strapped in the Biodex chair, and their tested arm was manually held in the standardized configuration because the system was not designed to withstand maximum joint torques. The maximal activity of the biceps and triceps brachii muscles was measured by requesting the participant to generate their maximal isometric elbow flexion and extension torque while the experimenter maintained the participant in the standardized configuration. Maximal voluntary torque generation in each direction was repeated twice. While this method is conventional for precisely measuring weakness, it is limited in that the maximum EMG magnitude obtained does not fully reflect the true maximum activation capacity of the paretic muscle. Methods such as twitch interpolation estimate voluntary activation capacity that is understood to be reduced in upper extremity (Garmirian et al., 2018, 2019) and lower extremity (Klein et al., 1985; Miller et al., 2009) muscles of individuals with stroke. Twitch interpolation, however, was beyond the scope of this study so max EMG during maximum voluntary torque was deemed sufficient to provide a frame of reference for the magnitude of the quiescence threshold discussed below.

### 2.4 Data analysis

Torque, angular position, and EMG data were analyzed. To begin, data corresponding to each angle of both the flexion and extension movement directions were extracted. These data segments were 1‐s time windows obtained 3 s after the arm was held still at each angle. This data segment was chosen as it was prior to the physical placement of the ultrasound probe. Data for the 90° and 150° angles were not collected for one participant due to discomfort in the device at these positions. In addition, data were not available for the non‐paretic arm of two participants.

To ensure that the measured responses were musculoskeletal, and not neurological, in nature, within each data segment, we confirmed that motor units were not active by identifying muscle quiescence. This was achieved by comparing the EMG signals from each data segment to baseline EMG activity. Baseline EMG activity was identified based on data collected at the biceps brachii and triceps brachii during a trial of 5 s when the subject was instructed to remain relaxed following the standardized stretch reflex habituation procedure. The EMG data were pre‐processed by first subtracting the mean amplitude so that the average baseline activity was of 0 mV. Subsequently, the data were rectified to permit extraction of the magnitude. The threshold baseline activity was defined as the sum of the mean plus three standard deviations of the pre‐processed baseline quiescent EMG signal (Rose, 2014). For each extracted and analyzed data segment, quiescent muscle activation was achieved if the mean activity of the pre‐processed signal was less than the baseline threshold. If the mean amplitude of the EMG signal for either the biceps brachii or triceps brachii exceeded the baseline threshold, then data for the corresponding participant, arm, angle, and movement direction combination would be discarded. For all the subjects, muscle activity for all trials was under this threshold such that no trials were discarded. It is still possible that undetectable muscle activation was present despite implementing this conventional approach to confirm the absence of increases in baseline muscle activity.

For each data segment, the main outcome measure was the mean torque. We visually inspected and analyzed all the data (e.g., angular position, angular velocity, angular acceleration) to ensure that the data extracted corresponded to no movement at the arm.

Post‐processing of the ultrasound data was performed with custom software (Lee et al., 2015) that extracts the shear wave velocity and quality factor, producing two 60 × 60 matrices for the region of interest (ROI, i.e., blue box in Figure 3). The company's proprietary software provides a “quality factor” value to indicate the “quality” of each shear wave velocity measurement. This is related to the cross‐correlation algorithm that the company software uses to track the propagation of shear waves through the tissue. Mean shear wave velocity was then calculated from the velocity values within the ROI that surpassed the device‐default 0.7 quality factor for each of the five trials within each of the seven joint angular positions.

**FIGURE 4:**
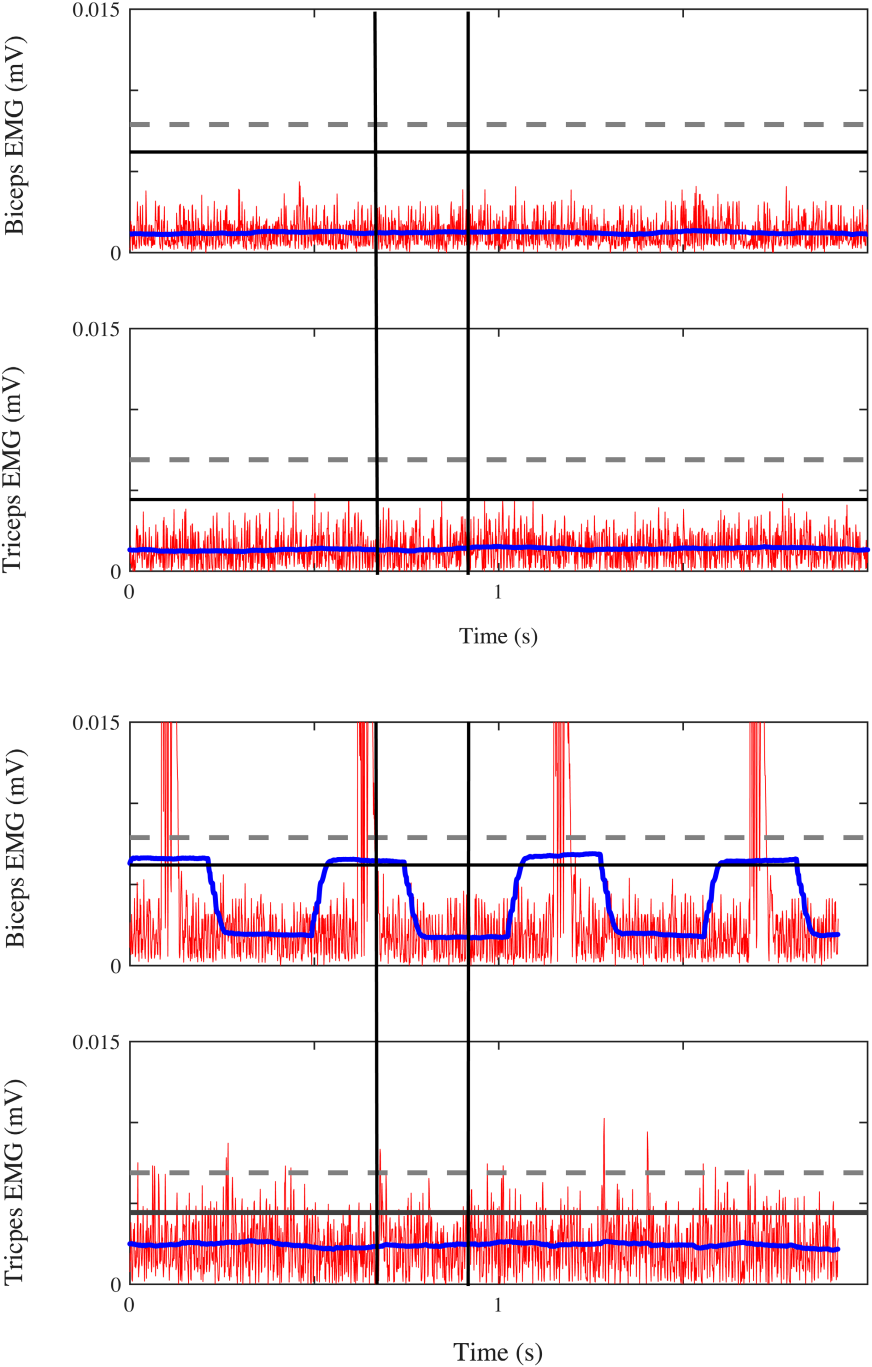
Example EMG data illustrating the post‐processing threshold‐based confirmation of muscle quiescence. EMG signals corresponding to a 2 s time frame of interest are displayed. Two black vertical lines depict 200 ms surrounding the ultrasound image capture. Significant muscle activation is not detected in either example for the biceps or triceps despite the presence of the acoustic ultrasound artifact. Processed EMG data (DC‐offset correction and full‐wave rectification) are indicated in red, processed EMG data (zero‐phase 250 ms moving window filter) in blue, and EMG quiescence threshold in black (horizontal line). For a greater perspective of the EMG magnitude, a threshold representing 3% maximum voluntary activation is indicated with a horizontal dotted line (gray).

Finally, the EMG signals that corresponded with each ultrasound image were analyzed for the 200 ms window prior to capturing the image to confirm the absence of muscle activity that would impact shear wave velocity. This process was similar to that mentioned above for confirmation of quiescence during the 1‐s torque data segment. In brief, images were discarded if either of the two filtered EMG signals were above the baseline threshold. An example of EMG data corresponding to ultrasound image capture is illustrated in Figure 4. Occasionally, there was a signal artifact from the ultrasound transducer (1 Hz) that occurred when the ultrasound transducer was close to the EMG sensor. EMG data were visually inspected to ensure that trials were not rejected due to the presence of the artifact during the 200 ms window. Instead, a broader window of 2 s was used for visualization to gain insight from the preceding and proceeding EMG signals surrounding the image capture window. The image was discarded only if it was determined that the source of the increased EMG signal was from muscle activation and not the transducer artifact. Of the entire data set, only 8 of ~700 images were rejected due to muscle activation.

### 2.5 Statistical analysis

We determined whether the arm, angular position, or their interaction significantly affected shear wave velocity and joint torque. The data were fit to a linear mixed‐effects model with participant as a random effect, and an analysis of variance identified significant effects. If a significant effect (*p* < 0.05) of angular position was identified, post‐hoc pairwise comparisons were conducted that accounted for the multiple comparisons by adjusting the *p*‐values using the Tukey method. A Pearson correlation coefficient assessed the relationship between joint torque and shear wave velocity for the paretic arm, non‐paretic arm, and both arms combined. Each correlation assessment was performed for the combined data set of all elbow positions.

## 3 RESULTS

**FIGURE 5:**
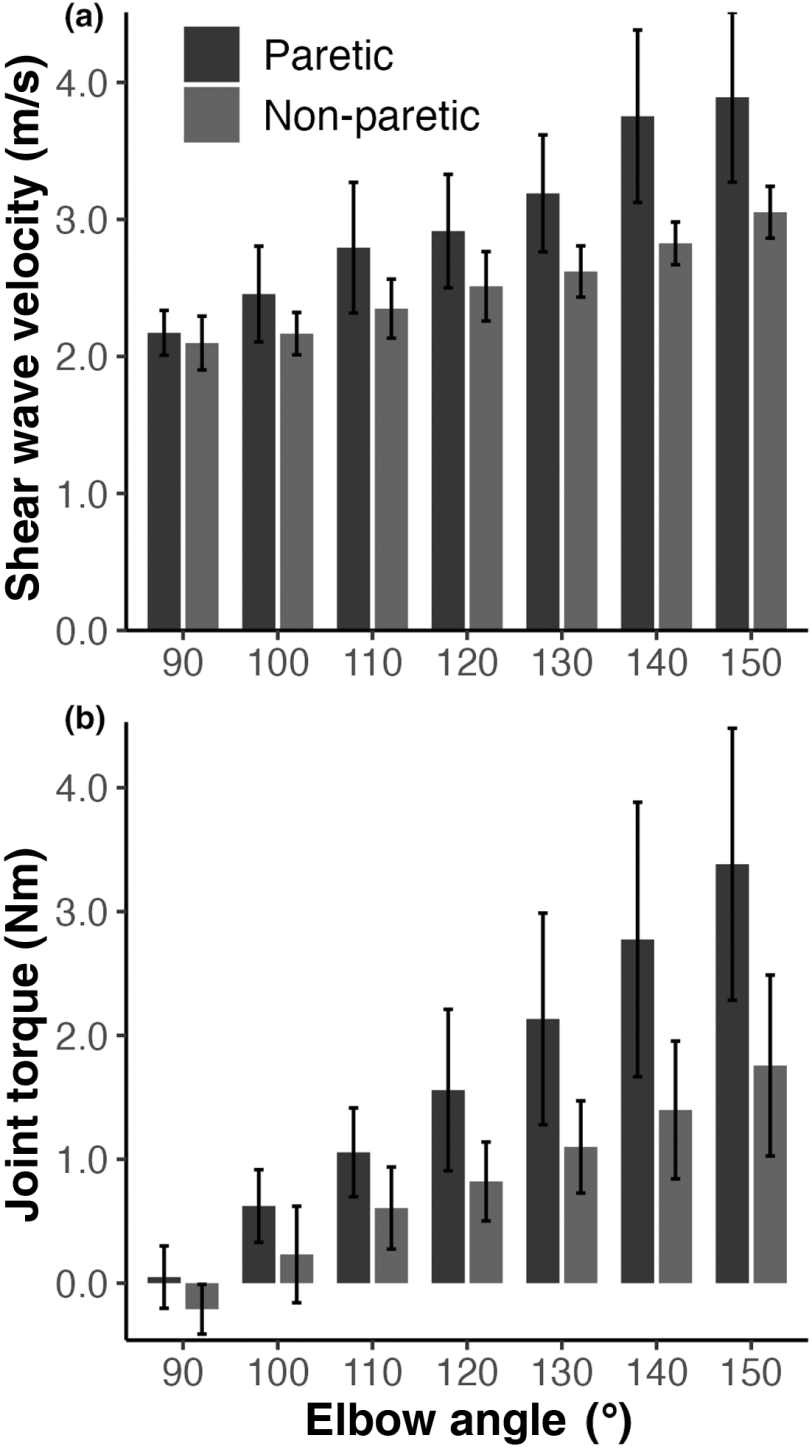
Mean shear wave velocity (a) and joint torque (b) with 95% CI error bars for the seven tested joint angles (90°–150°). The analysis of variance from the linear mixed‐effects model found significant effects (*p* < 0.05) of arm and angle for (i) shear wave velocity and (ii) joint torque. Pairwise comparisons indicated that a minimum of 30° was needed to obtain a significant difference (*p* < 0.05) between measurements for each of joint torque and shear wave velocity.

Overall, there were significant differences between the paretic and non‐paretic arms. The paretic arm had a greater shear wave velocity (F_(1,102)_ = 62.45; *p* < 0.001; effect size, η ^2^_*p*_ = 0.37) and joint torque (F_(1,102)_ = 37.59; *p* < 0.001; effect size, η ^2^_*p*_ = 0.26) than the non‐paretic arm (Figure 5a,b). The mean and standard deviation of the joint torque across all participants for the paretic arm was 1.63 ± 1.49 Nm and 0.82 ± 0.88 Nm for the non‐paretic arm, representing a 100% increase. An overall increase of 20% was found for shear wave velocity.

A greater elbow joint angle resulted in an increased joint torque (F_(6,102)_ = 22.38; *p* < 0.001; effect size, η ^2^_p_ = 0.57) and shear wave velocity (F_(6,102)_ = 27.94; *p* < 0.001; effect size, η ^2^_p_ = 0.62). Pairwise comparisons indicated that a minimum of 30° was needed to obtain a significant difference in measurements for the joint torque and shear wave velocity.

**FIGURE 6:**
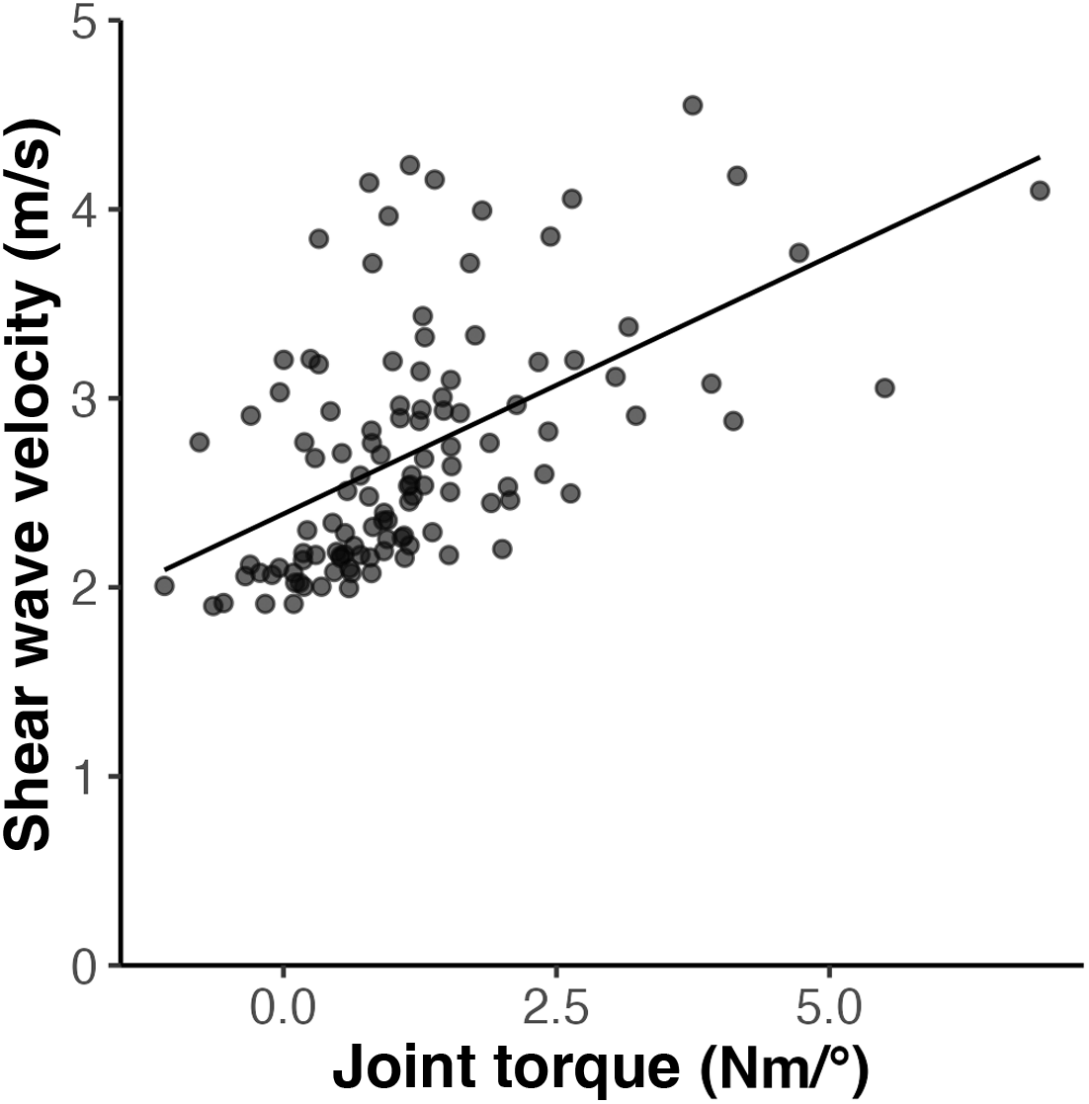
Shear wave velocity versus joint torque for the entire data set, illustrating the data spread. A best‐fit line is indicated for visual appreciation only.

Shear wave velocity increased as a function of joint torque (Figure 6). There was a significant and moderate correlation of shear wave velocity (*r* = 0.55, *n* = 119, *p* = 1.52e^−10^) with joint torque for the combined 90°–150° data set of both arms. There was also a moderate correlation between joint torque and shear wave velocity for the paretic (*r* = 0.49, *n* = 63, *p* = 5.17e^−5^) and non‐paretic (*r* = 0.52, *n* = 56, *p* = 3.45e^−5^) arms, individually.

## 4 DISCUSSION

The goal of this study, to demonstrate the criterion validity of ultrasound elastography for assessing muscle‐level changes following stroke, was primarily motivated by the need for a high‐resolution precise proxy for the clinical assessment of passive joint torque. The mechatronic measurement of joint torque, a global measure of resistance to passive angular joint motion, is a precise analog of current clinical manual assessment of joint mobility; however, muscle and specifically its connective tissue (i.e., the extracellular matrix, ECM) is assumed to be the greatest contributor to joint‐level resistance during passive motion (Lieber et al., 2017). Previous work showing the relationships between shear wave velocity, force, and stiffness in muscle (Bernabei et al., 1985; Eby et al., 2013) suggests using shear wave ultrasound elastography may provide a more objective assessment of the underlying mechanisms of altered joint mechanics in individuals with stroke‐related impairments. Our hypothesis was supported in that there was a relationship between joint torque and shear wave velocity in both paretic and non‐paretic limbs.

We interpret the moderate *r*‐values as evidence for the *criterion* validity of ultrasound elastography despite falling just under the 0.60 threshold recommended for support of validity of measurement in rehabilitation (Post, 2016). Post (2016) argues that *r*‐values under 0.60 raise questions of validity and that studies should be better designed with more similar criterion measures expected to result in higher correlation coefficients. A very similar metric to shear wave elastography is magnetic resonance imaging elastography. This, however, was not the goal of the present study. Instead, we were interested in the relationship with resistance to joint position (i.e., muscle length) and motion for which muscle tissue is the primary contributor. The ideal criterion, therefore, was a mechatronic measurement of joint torque. These metrics are clearly divergent but still thought to capture the same phenomena and were therefore expected to correlate but result in “moderate” *r*‐values at best. We do not believe a better criterion than joint torque exists and therefore stand by the results of the present study serving as evidence for criterion validity of shear wave elastography as a precise proxy for the clinical measurement of passive joint torque.

When comparing the limbs of individuals with stroke, the present study was consistent with previous reports of ultrasound‐based biceps metrics (Eby et al., 2016; Lee et al., 2015; Wu et al., 2017) and passive elbow flexion torque measures (Eby et al., 2016; Li et al., 2017) in the paretic limb. Under the assumption of muscle quiescence, the present study found a 20% overall mean increase in shear wave velocity for the paretic arm. In comparison, Wu et al. (2017) reported a 12%–19% increase in the resting paretic biceps. Lee et al. (2015) reported a maximum of 69% increase in shear wave velocity in resting paretic biceps. The discrepancy in shear wave velocities reported in these two studies may represent differences in sample populations with the present study. In Wu et al., a cohort of 9 of 31 individuals with stroke was identified as having negligible differences between arms, comparable to controls yet reflecting values obtained in the present study. In the Lee et al. study, four of 16 reported participants had two‐ to threefold increases in shear wave velocity on the paretic side. The remaining participants in the Lee et al. study reflected differences subjectively similar to the sample reported in the present study. The range of differences across studies reflects the natural heterogeneity of stroke presentation. Individuals with more severe functional impairments, as reflected by lower Fügl‐Meyer scores, tended to have greater differences in shear wave velocity between the paretic and non‐paretic side (Lehoux et al., 2020). An alternative explanation for a discrepancy across studies would be the implementation of a standardized reflex habituation procedure employed in the present study. As discussed in the methods, 20 fast (120°/s) elbow extension‐flexion stretches were conducted to habituate unwanted biceps stretch‐reflex activation (Patterson et al., 2022; Schmit et al., 2000) as undetected muscle activation stands to have a substantial impact on ultrasound elastography metrics. In fact, shear modulus is highly sensitive to muscle activation and capable of differentiating activations of even 3% and 7% maximum voluntary activation (Nordez & Hug, 2010), which is why the 3% threshold is indicated in Figure 4. To minimize undetected muscle activation as a source of error to the internal validity (Slack & Draugalis, 2001) of the experiment, the present study went beyond conventional visual EMG inspection by employing a precise threshold‐based confirmation during post‐processing to confirm quiescence.

The increase in shear wave velocity and joint torque on the hemiparetic side may be affected by changes in architecture, specifically, fascicle length. Decreased length in fascicles has been reported in muscles of the paretic arm (Li et al., 2007; Nelson et al., 2018), which would result in the passive portion of the length‐tension relationship shifting leftward similar to findings in the ankle by Gao et al. (2009), such that for a given joint angle, a larger increase in muscle tension would result. The methods employed in the present study use standardized joint angular position for measurement. Therefore, it is possible that the paretic biceps brachii was positioned farther along its operational curve in comparison to the non‐paretic side. Future investigations would benefit from standardizing by muscle fascicle length, perhaps with the assistance of extended view ultrasonography (Nelson et al., 2016), to more accurately account for stroke‐related changes such as shortened fascicle length (Nelson et al., 2018) and reduced number of sarcomeres (Adkins et al., 2021).

A compounding mechanism that could also be contributing to increases in shear wave velocity and joint torque in the paretic limb is a change in the amount or type of titin (Magid & Law, 1985) expressed within the myofibril and/or increases in the deposition of collagen (Meyer & Lieber, 2011) in the ECM. While the exact roles of titin and collagen in passive muscle tension are still debated (Lieber et al., 2017), it is widely excepted that the parallel elastic component contributes to the passive tension within the Hill‐type model. Recent evidence reporting echogenicity in B‐mode imaging supports alterations in muscle composition following stroke (Lee et al., 2015). Although it is unclear whether fascicle length and/or connective tissue changes explain the differences between limbs following stroke, it does not detract from the study objective of evaluating the *criterion* and *construct* validity of ultrasound elastography in the paretic biceps brachii that was further supported by a positive direct correlation between ultrasound and torque measures and detection of a difference between limbs on both metrics.

Interestingly, a relatively larger difference was observed in joint torque than shear wave velocity between the paretic and non‐paretic sides. Joint torque was 100% greater on the paretic side than on the non‐paretic side whereas shear wave velocity was 20% greater on the paretic side. The larger torque difference may represent the summative effects of all muscles/tissues crossing the elbow joint that contribute to the joint torque measure. This includes the ECM and tendons of elbow flexor and extensor muscles, joint capsules, and other tissues. In this study, shear wave velocity was only measured in the biceps brachii. The other elbow flexors and extensors making up a significant proportion of the cross‐sectional area may undergo similar changes.

More precise tools to evaluate changes in passive muscle mechanical properties are necessary for the advancement of current clinical methods. Clinical assessment of passive muscle properties is limited to manual palpation, joint mobility, and muscle length testing (Reese et al., 2010). Passive movement assessments can also be compounded by reflexive muscle activation, such as with resistance to lengthening due to abnormal stretch reflex, or spasticity (Bohannon & Smith, 1987). Importantly, manual assessments of spasticity cannot differentiate resistance due to passive intrinsic muscle properties and due to reflexive activation as is possible with mechatronic devices (Mirbagheri et al., 2007). One commercially available instrument, Myotonometer (Neurogenic Technologies, Inc), is capable of measuring tissue deformation, capturing muscle tissue properties (Chuang et al., 2012; Leonard et al., 2001, 2006) with established reliability (Chuang et al., 2012); however, it is limited in application in individuals with more adipose tissue and accounting for fat deposition is important when evaluating muscle tissue post‐stroke (Adkins et al., 2021). In this respect, shear wave ultrasound elastography may apply to a greater proportion of the stroke population. In stroke rehabilitation, the ability to quantify muscle mechanical properties would provide clinicians with a powerful tool to prioritize the underlying factors of movement problems. Pathological changes in the passive mechanical properties of muscle will only compound abnormal neuromuscular performance impairments, such as reduced reaching range of motion that occurs as a function of abduction loading due to the expression of the flexion synergy (Ellis et al., 2008, 2016; Sukal et al., 2007). Quantifying changes in muscle through the implementation of shear wave ultrasound elastography will identify new therapeutic targets, precipitating the expansion of conventional approaches for ameliorating movement dysfunction in stroke recovery.

## 5 LIMITATIONS

While exquisite attention was devoted to the confirmation of muscle quiescence, it is possible that undetectable flexor activation was occurring, especially toward the end of range of motion due to hyperactive stretch reflexes resulting from motoneuron hyperexcitability. If so, this would have been reflected in all metrics and enhanced on the paretic side, additionally reflecting active as opposed to solely passive/intrinsic joint torque. While it is a limitation for the comparison of arms, it is not a limitation for the primary evaluation of criterion validity in that, if there is undetectable muscle activation, it is present in both ultrasound and joint torque measurements. Other methods including ischemia (Lorentzen et al., 2010), phenol motor point block (McCrea et al., 2004), brachial plexus blockade (Neal et al., 2009), or sleep atonia (Arrigoni et al., 2016) may fully suppress muscle activation and resolve this limitation in future research.

It should be acknowledged that passive elbow torque is the result of a combination of muscles acting about the elbow. Contributions from some muscles, like biceps brachii, to passive joint torque will vary with joint position as it has a decreasing moment arm length with elbow joint extension beyond ~90° (Akagi et al., 2012; Amis et al., 1979). Despite evaluating solely biceps brachii, shear wave values were correlated with passive joint torques and were able to differentiate arms of individuals with hemiparetic stroke. Future work may employ imaging to quantify in vivo participant‐specific moment arms, muscle cross‐sectional area, and pinnation angles as a function of joint angular position (Akagi et al., 2012, 2014; Langenderfer et al., 2004; Murray et al., 1995) to help determine the biceps' contribution to the overall passive elbow torque.

The most acute joint angle measured at the elbow joint was 90°. The biceps brachii has a reported slack angle of 95° (Lacourpaille et al., 2013) suggesting that the first position of measurement may have been reduced due to the biceps being slack. This does not appear to have had a large impact due to the statistical analysis demonstrating a difference between joint positions of at least 30° regardless of position.

Finally, the protocol originally intended to also measure shear wave velocity in the lateral head of the triceps. However, imaging of the triceps was dropped from the protocol following the inability to meet criteria #3 in ultrasound methods above (velocity data throughout the region of interest) in the first five participants.

## AUTHOR CONTRIBUTIONS

Ellis was involved in project oversight, scientific conceptualization, protocol development, data collection, statistical analyses, manuscript drafting/editing. Gurari scientific conceptualization, robotic protocol development, robotic device, and ultrasound interface and protocol implementation, development of robotic device data processing routines, statistical analyses, manuscript drafting/editing. Gerritsen was involved in robotic device and ultrasound protocol implementation, data collection, manuscript drafting/editing. Lee was involved in scientific conceptualization, ultrasound training, protocol development, and implementation, development of ultrasound data processing routines, manuscript editing. Wang was involved in development of robotic device data processing routines, statistical analyses, manuscript drafting/editing. Dewald was involved in Initial scientific conceptualization, protocol development, analysis approach, manuscript editing.

## FUNDING INFORMATION

This work was supported by the National Institute of Child Health and Human Development R01 Grants (HD84009: PIs—Dewald/Murray, HD096071: PI—Ellis), the National Institute of Neurological Disorders and Stroke R01 Grant (NS105759: PI—Dewald), and the National Institute of Child Health and Human Development K25 Grant (HD096116; PI—Gurari).

## CONFLICT OF INTEREST STATEMENT

None of the authors have financial or other relationships that might lead to a perceived conflict of interest.
